# Acute Primary Adrenal Insufficiency after Hip Replacement in a Patient with Acute Intermittent Porphyria

**DOI:** 10.1155/2018/2353172

**Published:** 2018-01-04

**Authors:** Adele Latina, Massimo Terzolo, Anna Pia, Giuseppe Reimondo, Elena Castellano, Micaela Pellegrino, Giorgio Borretta

**Affiliations:** ^1^Division of Endocrinology, Diabetology and Metabolism, Santa Croce e Carle Hospital, Via M. Coppino 26, 12100 Cuneo, Italy; ^2^Internal Medicine 1, Department of Clinical and Biological Sciences, University of Turin, San Luigi Gonzaga Hospital, Regione Gonzole 10, Orbassano, 10043 Turin, Italy

## Abstract

Adrenal insufficiency is a potentially life-threatening condition when it occurs acutely, as in adrenal hemorrhage. Generally it is not reversible and requires chronic replacement therapy. Acute intermittent porphyria (AIP) is a rare genetic disease characterized by alterations in heme biosynthesis that result in accumulation of precursors in tissues. A crisis can be triggered by many conditions such as surgery and infections. Symptoms are similar to those of acute hypoadrenalism. Moreover, both conditions are characterized by hyponatremia. We describe the case of a postmenopausal woman known to be affected by AIP who developed after surgery a primary adrenal insufficiency associated with adrenal enlargement; the latter completely reverted in six months.

## 1. Introduction

Adrenal insufficiency is an infrequent endocrine disorder mostly due to autoimmune adrenalitis; less common causes are infections, trauma, and metastatic cancer. Adrenal insufficiency is a potentially life-threatening condition when acute, as in adrenal hemorrhage, necrosis or thrombosis. Generally, adrenal damage is not reversible and requires chronic replacement therapy.

Transient adrenal insufficiency has been described as a manifestation of viral, parasitic, or mycotic infection, in particular in premature newborn infants or immunocompromised patients.

Acute intermittent porphyria (AIP) is a rare genetic disease characterized by alterations in heme biosynthesis which result in the accumulation of precursors in tissues. An AIP crisis can be triggered by many conditions such as surgery, infections, fasting, and the use of many drugs including anesthetics. The symptoms of a crisis are abdominal pain, nausea, and vomiting, similar to those of acute hypoadrenalism. Both conditions are also characterized by the development of hyponatremia and, in biochemically active AIP, cortisol levels have been reported to be reduced; thus the diagnosis may be challenging.

We describe the case of a postmenopausal woman known to be affected by AIP who after surgery developed a primary adrenal insufficiency associated with adrenal enlargement, which completely reverted in six months. She was initially treated with replacement therapy which was subsequently reduced in dose, obtaining basal adrenocorticotrophic hormone (ACTH) and cortisol normalization, while maintaining an inadequate cortisol response to the ACTH test.

## 2. Case Presentation

A 65-year-old woman was referred to the intensive care unit under suspicion of a postoperative AIP crisis.

She had a history of previous surgery for breast fibroadenoma and was chronically affected by colonic diverticulosis, chronic gastritis, hypertension, and mild depressive syndrome. She was treated with a proton-pump inhibitor, metoprolol, enalapril, acetylsalicylic acid, and trazodone.

Porphyria had been diagnosed one year previously, after demonstration of porphobilinogen and aminolevulinic acid increase during an episode of abdominal pain, vomiting, and hyponatremia. During the same hospitalization, because of the onset of chest pain, ECG alterations, and troponin increase, the patient underwent a coronary angiography which was negative for stenosis. Thus takotsubo, a stress cardiomyopathy simulating an acute coronary syndrome, was diagnosed. In this critical care context, an ACTH and cortisol assay were performed, resulting in 4.0 pM/L (n.v. 2–14) and 998.8 nM/L (n.v. 165.5–507.7), respectively.

Nine days after a right hip replacement surgery due to severe arthrosis, the patient developed nausea and vomiting, abdominal pain with paralytic ileus, urinary retention, and upper limb paresthesia.

Laboratory analysis (see Table 1) showed normochromic anemia, a platelet count in the lower range, prolonged partial thromboplastin time, elevated C-reactive protein and procalcitonin, hypokalemia, and hyponatremia. Thus, considering the symptoms, an acute AIP crisis was suspected.

A chest X-ray was taken, which showed focal pneumonia with bilateral pleural effusion.

The patient started treatment for AIP with hemin and glucose solution, in addition to antibiotic treatment.

Due to the persistence of severe hyponatremia (123.8 mmol/L) and symptoms such as asthenia and nausea 19 days later, hypoadrenalism was suspected. The ACTH was 43 pM/L and cortisol 163.3 nM/L (data confirmed by a second sample) with PRA 0.3 *μ*g/L/h (normal values: 0,60–4,18; RIA Immunotech Beckman Coulter), aldosterone 47.2 pmol/L (normal values: 48,3–270,0; RIA Immunotech Beckman Coulter), and DHEA-S < 0.4 umol/L (CLIA, Centaur XPT, Siemens). The patient was treated with no drug interfering with adrenal function. The ACTH test 250 *μ*g iv showed no response of cortisol (see Table 2).

**Figure 1:**
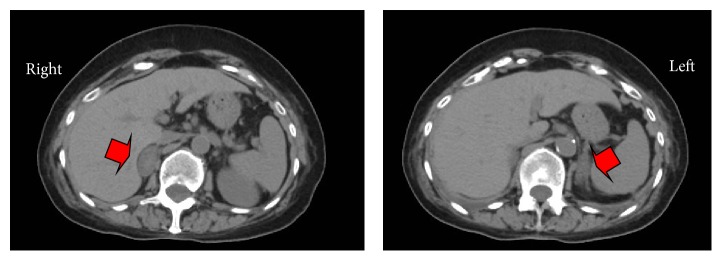
Abdomen CT showing bilateral adrenal enlargement, highlighted by red arrows, with a hyperdense central area of 2 cm of maximum diameter (HU 25–35) in right adrenal gland.

An abdominal computed tomography (CT) showed bilateral adrenal enlargement, with a hyperdense central area of 2 cm maximum diameter (HU 25–35) in the right adrenal gland (Figure 1). No suspicious lesion was identified.

Anti-adrenal antibodies were undetectable, as were anticardiolipin antibodies.

A tuberculin test was negative and so were tests on candida and aspergillus antigens. CMV DNA was undetectable. Anti-HIV and anti-treponema antibodies were also negative. Other mycosis infections (cryptococcosis, histoplasmosis, and coccidioidomycosis) and parasitoses (trypanosomiasis) were ruled out through appropriate serological tests.

The patient was initially treated with oral cortisone acetate, 25 mg in the morning and 12.5 mg in the afternoon, with rapid improvement of symptoms and normalization of sodium levels. The patient was then discharged on glucocorticoid replacement therapy (cortisone acetate 12.5 mg twice a day). Diagnosis of primary hypoadrenalism of uncertain etiology associated with bilateral adrenal enlargement was confirmed.

Three months later, the basal cortisol was 149.0 nM/L which did not increase (see Table 2) after the ACTH test (24 h after cortisone acetate withdrawal). Basal ACTH was 71 pM/L, PRA 1.0 *μ*g/L/h, aldosterone 322.2 pmol/L, and DHEA-S < 0.4 umol/L.

**Figure 2:**
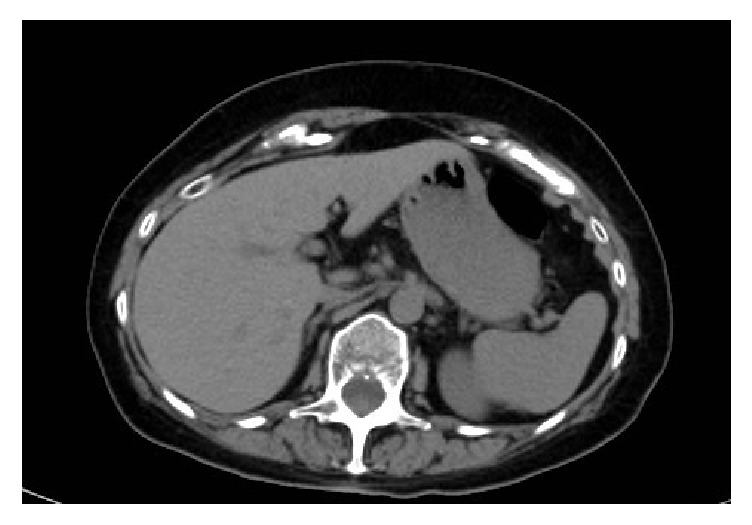
Abdomen CT showing adrenal glands normalized in volume 6 months later.

In a CT repeated 6 months later, adrenal glands presented with normalized volume (Figure 2), and, in particular, the right lesion was no longer detected.

One year later, after gradual reduction in the dose of cortisone acetate (6.25 mg twice a day and then 6.25 mg once a day), ACTH and basal cortisol had normalized (see Table 2). However the cortisol remained nonresponsive after ACTH, with PRA and aldosterone always normal and DHEA-S unchanged.

At present (30 months after diagnosis) the patient is continuing a minimal dose of cortisone acetate (6.25 mg once a day) without symptoms. The last ACTH was 18 pM/L with normal PRA and aldosterone. Cortisol basal levels remain in the normal range and are progressively increasing (see Table 2), although there has still been no response of the cortisol to the ACTH test. At CT examination the left adrenal gland was of normal volume, while the right one showed an apparent reduction in size with an inhomogeneous structure. These recent data support the progressive recovery of the glucocorticoid secretion, although with an incomplete functional normalization. Subsequent follow-up will assess whether the partial deficit is persistent or if definitive recovery is possible.

## 3. Discussion

Adrenal insufficiency is a rare condition mostly due to autoimmune disease but potentially developing after trauma, infections, neoplastic infiltration, or vascular damage, which generally requires chronic replacement therapy.

Transient adrenal insufficiency on the other hand is infrequent and is reported in the literature in premature newborn, in infective diseases, in survivors of critical illness [1], or, rarely, following acute adrenal hemorrhage [2]. Such a hemorrhage is a severe potentially life-threatening or even lethal condition when massive and bilateral. The clinical diagnosis is challenging. This is particularly the case in critically ill patients, due to nonspecific symptoms and signs such as abdominal pain, vomiting, fever, hypotension, and altered conscious state. Moreover a limited adrenal hemorrhage may give nonspecific symptoms. Hemorrhage has been described after surgery, such as cholecystectomy, total knee arthroplasty, duodenopancreatectomy, and vertebral surgery, in sepsis, and is rarely reported as a spontaneous event, mostly in pregnancy and in particular conditions such as polycythemia vera and antiphospholipid syndrome.

In our patient, who experienced an acute primary hypoadrenalism with bilateral glandular enlargement, the CT appearance and subsequent partial normalization of the adrenal function could be consistent with a bilateral congestion leading to a hemorrhage, more evident in the right adrenal gland, which had presumably occurred 1-2 weeks before the imaging.

The history of AIP delayed the diagnosis of hypoadrenalism due to the development of symptoms suggesting a postoperative crisis. This delay may explain the CT appearance of the adrenals, which was not typical of a hemorrhage [3], though the hyperdense central area could be due to the outbreak where the greatest concentrations of hemoglobin were localized.

In our patient adrenal insufficiency seems to be a consequence of recent surgery, which can potentially induce adrenal hemorrhage, by itself but in particular due to the heparin treatment, which is routinely used in the postoperative period. The cortisol increase which had been observed during a critical stress condition (takotsubo cardiomyopathy) presumably indicates normal adrenal function, at least one year before. Autoimmune and infective hypoadrenalism were ruled out. Risk factors for adrenal hemorrhage were present such as postsurgery heparin treatment (which could explain the platelet count in the lower range and the prolonged partial thromboplastin time) and the occurrence of septic condition, which may have worsened the clinical status. Undetectable anticardiolipin antibodies, which are the most sensitive in diagnosing antiphospholipid syndrome [4], allow reasonably excluding this cause of adrenal failure.

The hypothesis of transient adrenal insufficiency related to a poor entity hemorrhage is consistent with the clinical evolution of the patient who reverted to normal adrenal CT presentation in 6 months and to almost normal basal function in 12 months. However, the lack of cortisol response to ACTH test suggests that cortisol secretion could not be further stimulated and therefore that the adrenal functional recovery was partial.

AIP patients present similar symptoms to those of acute hypoadrenalism (abdominal pain, nausea, and vomiting) thus resulting in a potential confounder for clinicians. To the best of our knowledge only two cases of acute hypoadrenalism have been reported after hip replacement. The first was subsequent to enoxaparin-induced thrombocytopenia [5], and the second was in a 75-year-old woman treated after surgery with dabigatran who underwent thrombolysis for massive pulmonary embolism [6]. In both cases, adrenal insufficiency was definitive.

Our case highlights how difficult the differential diagnosis is in AIP patients, in whom adrenal insufficiency can be misdiagnosed because of similar manifestations. A cause-effect relationship between AIP, major surgery, and hypoadrenalism is conceivable but it cannot be proved: these conditions could be coincident even if independent.

However, acute hypoadrenalism represents a potential life-threatening condition which needs to be taken into account, especially when severe and persistent hyponatremia is observed.

## Figures and Tables

**Table 1:** Biochemical data nine days after hip replacement.

		Reference values
RBC (×10^6^/uL)	2.96	4.2–5.4
Hb (g/dL)	9.0	12.0–16.0
MCV (fl)	88.9	77–94
PLT (×10^3^/uL)	150	150–400
aPTT (sec.)	47.2	25–40
CRP (mg/L)	215	<10
PCT (ng/mL)	0.305	<0.046
Na (mmol/L)	132.6	137–145
K (mmol/L)	3.0	3.6–5.0

**Table 2:** Adrenal function before hip replacement and during follow-up.

	Time with respect to hip replacement					
	One year before	19 days after	21 days after	3 months after	12 months after	30 months after
ACTH (pM/L) n.v. 2–14	4	43		71	11	18
Basal cortisol (nM/L) n.v. 165.5–507.7	998.8	163.3	278.7	149.0	366.9	477.3
Cortisol 30′ after ACTH 250 *µ*g iv			278.7	162.8	366.9	460.8
Cortisol 60′ after ACTH 250 *µ*g iv			264.9	154.5	331.1	502.1

## References

[1] Nylen E. S., Muller B. (2004). Endocrine changes in critical illness. *Journal of Intensive Care Medicine*.

[2] Vella A., Nippoldt T. B., Morris J. C. (2001). Adrenal hemorrhage: a 25-year experience at the Mayo Clinic. *Mayo Clinic Proceedings*.

[3] Lykissas M. G., Galanis S. H., Borodimos A. C., Pakos S. G. (2006). CT diagnosis of acute adrenal insufficiency due to bilateral adrenal haemorrhage. *European Journal of Radiology Extra*.

[4] Presotto F., Fornasini F., Betterle C., Federspil G., Rossato M. (2005). Acute adrenal failure as the heralding symptom of primary antiphospholipid syndrome: report of a case and review of the literature. *European Journal of Endocrinology*.

[5] Reverdy F., Freichet M., Grozel J.-M., Tassin C., Piriou V. (2013). Bilateral adrenal hemorrhage after heparin-induced thrombocytopenia, a rare cause of shock. *Annales Françaises d’Anesthésie et de Réanimation*.

[6] Best M., Palmer K., Jones Q. C., Wathen C. G. (2013). Acute adrenal failure following anticoagulation with dabigatran after hip replacement and thrombolysis for massive pulmonary embolism. *BMJ Case Reports*.

